# Community physiotherapy or community-based physiotherapy

**DOI:** 10.15171/hpp.2017.10

**Published:** 2017-03-05

**Authors:** Pavithra Rajan

**Affiliations:** ^1^Fellow, Shastri Indo-Canadian Institute, Canada; ^2^Unit 4K, 15 Campbell Street, Parramatta, New South Wales 2150 Australia

## Dear Editor,

The profession of physiotherapy gained recognition during the time of World War I,^1^ with services being rendered in acute-care as well as community settings. It can thus be inferred that physiotherapy services in and for the community could have begun as early as 1914. Since then, there has been ample research globally (developed world more than the developing) on this specialization of physiotherapy and the importance of the same need not be more emphasized. The terms “community physiotherapy” and “community-based physiotherapy” have been used interchangeably by many researchers, although the meaning of each is different. Based on the concepts discussed in the original paper^2^ and book on community-based nursing by Hunt,^3^ this paper hopes to highlight the differences between community physiotherapy and community-based physiotherapy.

In community physiotherapy, the emphasis is on the setting “where” the treatment takes place. As the name clearly suggests, it is physiotherapy services “in the community” as opposed to acute-care setting. The physiotherapist visits the patient in the community and offers treatment. Community could be the patient’s home or residential care facility where the patient resides. For instance, consider the case of a middle aged man admitted at a hospital for myocardial infarction. After completion of treatment, the patient returns home and is visited by the physiotherapist for further treatment and management. This is a classic example of community physiotherapy. Due to advances in technology and health care services, community physiotherapy could be easily incorporated into the long term management of patients in the community.

**Figure 1:**
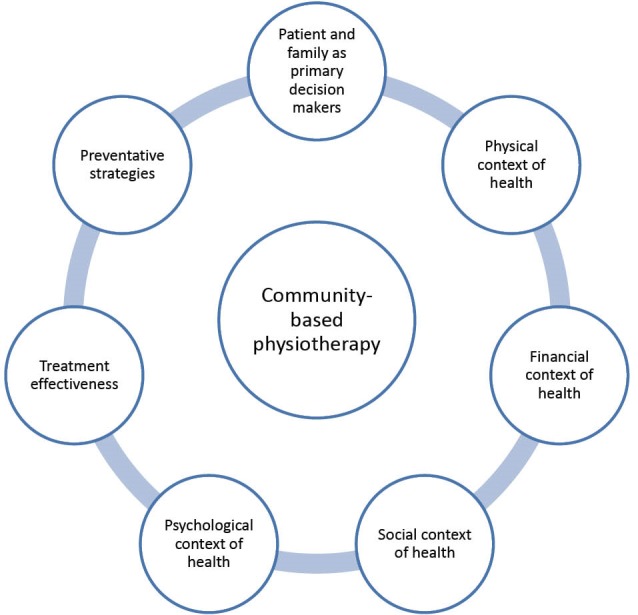
Community-based physiotherapy.

Community-based physiotherapy is “how” the treatment takes place. It has nothing to do with the “setting”, but is merely the way of practice. Community-based physiotherapy could be initiated at an acute –care setting. It can be explained using the following concepts (please refer to Figure 1):

1. The patient and caregivers (including family) are the primary decision-makers.
2. Holistic approach to treatment and management is undertaken, including physical, psychological, financial and social contexts.
3. Treatment effectiveness including preventative strategies is emphasized.

In today’s world of holistic approach to health care, the patient is treated by many health care professionals. Consider the earlier example of the middle aged man with myocardial infarction admitted in the intensive cardiac care unit (ICCU). During his stay in the ICCU, he is likely to be treated by the intensivist, cardiologist, nurse, physiotherapist, dietician and social worker. Once he is transferred to the wards, the health professionals attending to him might change, depending on the financial condition and preference of the patient and his caregivers (family). Occupational therapist might be included in the team. Once he is discharged, social worker, dietician, physiotherapist and occupational therapist might be delivering community services. There is changing number of health professionals at each stage (ICCU, ward and home) and community-based services might help in smooth transition from one phase to another. In the context of physiotherapy, involving the patient (if his condition permits) and his family and caregiver in the treatment strategies can ensure optimal continued delivery of health care. In addition, the treatment strategies might differ based on the financial and social domains. If the patient hails from a remote village, where physiotherapy services are negligible, the physiotherapist at the acute care setting might need to educate him and his family about after-discharge heart care. Further, the risk factors need to be studied in order to prevent another similar episode of heart attack. This could be extended to the assessment of his family and caregivers, to avoid such episodes in future. Community-based physiotherapy could be more progressive by providing handouts for prevention of myocardial infarction to be distributed by the patient, family and caregivers to the village residents. Thus, community-based physiotherapy can be started at the point of first contact with the patient, which is usually an acute-care setting.

## Ethical approval

None to be declared.

## Competing interests

Author declares that he has no competing interests.

## Author’s contribution

PR is the single author of the paper.
